# Effects of Surface Soil Removal on Dynamics of Dissolved Inorganic Nitrogen in a Snow-Dominated Forest

**DOI:** 10.1100/tsw.2001.311

**Published:** 2001-11-22

**Authors:** M. Ozawa, H. Shibata, F. Satoh, K. Sasa

**Affiliations:** Graduate School of Agriculture, Hokkaido University, Sapporo, Japan

**Keywords:** N dynamics, vegetation and surface soil removal, snowmelt, soil solution

## Abstract

To clarify the effect of vegetation and surface soil removal on dissolved inorganic nitrogen (N) dynamics in a snow-dominated forest soil in northern Japan, the seasonal fluctuation of N concentrations in soil solution and the annual flux of N in soil were investigated at a treated site (in which surface soil, including understory vegetation and organic and A horizons, was removed) and control sites from July 1998 to June 2000. Nitrate (NO3) concentration in soil solution at the treated site was significantly higher than that of the control in the no-snow period, and it was decreased by dilution from melting snow. The annual net outputs of NO3 from soil at the treated site and control sites were 257 and –12 mmol m year, and about 57% of the net output at the treated site occurred during the snowmelt period. NO_3_ was transported from the upper level to the lower level of soil via water movement during late autumn and winter, and it was retained in soil and leached by melt water in early spring. Removing vegetation and surface soil resulted in an increase in NO_3_ concentration of soil solution, and snowmelt strongly affected the NO_3_ leaching from treated soil and the NO_3_ restoration process in a snow-dominated region.

## INTRODUCTION

Nitrogen (N) is an essential nutrient for the growth of terrestrial organisms[1]. The dissolved inorganic N in soil, which is available to vegetation, forms a small proportion of total N, and most forest ecosystems are N limited. Artificial disturbance of forest ecosystems, e.g., clear-cutting or whole tree harvests, often causes the increase in $NO_3^-$ concentrations of soil solution and drainage water[2,3]. A major factor of that is the decrease in vegetation uptake on N that provides more $NH_4^+$ to be available to the nitrifers[2,3]. Moreover, the changes of microbial effects, e.g., the increase in mineralization and nitrification due to the increase in soil temperature and moisture[4,5] and the decrease of microbial immobilization[6], are also important factors. In addition to these biogeochemical effects, there are hydrological effects on the fluctuation of dissolved inorganic N. Since $NO_3^-$ is mobile anion in soil, the hydrological movement into soil is very important for the fluctuation of $NO_3^-$ concentrations in soil solution. In a snow-dominated region, levels of $NO_3^-$ and other elements in forest soil and streamwater increase in early spring due to fast and heavy infiltration of snowmelt water into soil [7,8].

In Hokkaido, in northern Japan, snowfall is a significant component of annual precipitation, and the soil preparation of the removal of both covered vegetation (mainly bamboo grass) and surface soil was extensively implemented. This treatment is efficient for assistance of natural regeneration precluded by a thick cover of bamboo grass. It is different from clear-cutting in that it was removed from the forest floor and rooted soil, which was a major N pool of forest soil because it contains a large amount of organic matter, fine root, and a large number of microbes. The mineral soil existed below the root sphere and is less affected by vegetation uptake and microbial activity before treatment exposure. The increase in $NO_3^-$ concentration in such an exposed mineral soil was reportedly caused by the decreased microbial immobilization rate[6]. A similar pattern may appear in this study site. Such an intensive soil preparation would often concur with forest management; however, little is known about this.

In a natural, undisturbed Hokkaido forest, $NO_3^-$ leaching rarely occurs, although there is a large amount of essential soil output during the snowmelt season[9]. However, leaching large amounts of $NO_3^-$ from soil would occur if the soil is disturbed in such a snow-dominated forest. The objectives of this study were to determine (1) the concentration changes of dissolved inorganic N and other soil elements after removal of vegetation and surface soil and (2) the effects of snowpack and snowmelt on the $NO_3^-$ concentration and budget of such disturbed soil. Our approach was to measure inorganic ion concentrations in soil solution, rainfall, and snowpack on an annual basis—including the snowpack and snowmelt seasons—and to calculate fluxes in treated and control sites in Hokkaido.

## MATERIALS AND METHODS

### Site Description

The study was conducted in Uryu Experimental Forest of Hokkaido University (42°16′N,142°21′E) in northern Hokkaido. The altitude of the site is 320 m.a.s.l. There is no source of acidic pollutants near this region. The soil is Dystrochrept (Soil Taxonomy, USDA 1994), and the parent material consists of andesite of Tertiary Age. The O horizon is about 5-cm thick, the A horizon is about 0- to 10-cm deep, the AB horizon is about 10- to 25-cm deep, the B horizon is about 25- to 35-cm deep, and the C horizon appears under 35-cm deep. The overstory of the forest comprises about seven species: predominantly *Quercus crispula*, *Abies sachalinensis*, and *Acer mono Maxim.*, and most of the forest floor is covered by sasa bamboo grass (*Sasa senanensis*). This study was carried out from July 1998 to October 2000. The mean annual precipitation during the study period was converted from 1467 mm, 40% of which was supplied by snow (from the middle of November to early April). The mean annual temperature during the study period was 4.2°C. The soil was unfrozen even during winter due to the thermal insulation effect of the dense snow. We divided the observation period into three periods: snowpack period (from November to March the following year), snowmelt period (from April to the middle of May), and no-snow period (from the middle of May to October).

Three study plots—one treated plot (treatment plot: TR plot) and two control plots—were prepared for the study. In the TR plot, the vegetation and surface soil (20- to 30-cm thick) were removed in 1996, and mineral soil was exposed after treatment. The initial vegetation was sasa bamboo grass, and residues of bamboo attended with treatment did not remain and mixed in with the mineral soil. The population of regenerated vegetation seedlings was very small in 1998 and was five to ten plants per square meter in 1999 and 2000. The two control plots were located in wide open forest adjacent to the TR plot; one is thickly covered with sasa bamboo grass (CS plot), and the other is an area of natural mixed forest (CT plot). Each plot was one replication; the area of each plot was 5 × 10 m sited within 1 ha.

### Field Observation Method

Ceramic-cup lysimeters were installed in each plot for collecting soil solution. Two lysimeters were installed at 10 cm below the soil surface (upper level) and at 40 cm below the soil surface (lower level, beneath the rooted soil). In November 1998, the two lysimeters specially designed for winter were also installed in each plot and depth. The soil solution collected under the snowpack was stored in one bottle from the three lysimeters, and the bottle was buried in unfrozen soil[9]. The sample collection tube from the bottle was extended above the snowpack and wrapped with electric heating tape to prevent the soil solution from freezing during the sampling procedure. The operation of the heating tape was controlled by a thermistor, which was only turned on when the temperature fell below 4°C. Thermal insulation tape was wrapped around the heating tape to prevent artificial melting of the snowpack. Heating the sampling tube was done only during sample collecting. Rainfall was collected at an open site adjacent to the TR plot, and throughfall was collected in the CT plot using polyethylene funnels (each 30 cm in diameter) and bottles. Snowpack was collected using a cylindrical snowpack collector (5 cm in diameter). Samples were collected weekly in 1998 and 1999 and monthly in 2000 during the no-snow period. During the snowpack and snowmelt periods, snowpack and soil solution samples were collected one to three times monthly. Soil temperature was measured hourly with a thermo recorder (TR-71S, T&D Corporation).

### Chemical Analysis

All water samples were transported to the laboratory and filtered through a 0.2-µm membrane filter to measure the ionic concentrations of major inorganic cations ($Na^+$, $K^+$, $NH_4^+$, $Ca^{2+}$, and $Mg^{2+}$) and anions ($Cl^-$, $NO_2^-$, $NO_3^-$, and $SO_4^{2-}$) by an ion chromatography (DX500 chromatograph system, DIONEX Co. Ltd.). The pH values were measured using a glass electrode.

### Elemental Budget Calculation

The elemental budget in each plot was estimated from July 1998 to June 1999. The output of water from soil that was 40-cm deep and scarcely affected by vegetation uptake accounted to subtract the potential evaporation from the input water[9]. Potential evaporation was calculated using the Penman equation based on meteorological data observed at the site remote from the study plot about 5 km, and it was assumed to be zero during the snowpack and snowmelt periods. The amount of input water was estimated from the rainfall in the TR and CS plots, and from the throughfall in the CT plot during the no-snow period. During the snowpack period, the input water to the soil was estimated from the meltwater from the bottom of the snowpack in each plot; melting velocity was assumed to be 0.3 mm day$^{-1}$[10]. The total amount of input water in the snowmelt period was calculated as the sum of the snowpack water (collected on February 26, 1999, the day of maximum snow depth) and rainfall after the beginning of snowmelt. The elemental outputs from the soil were calculated from the amount of output water and mean ionic concentration of soil solution at 40 cm in depth. Input fluxes of soil elements were calculated from the amount of input water and mean ionic concentration of rainfall, throughfall, or snowpack during each period.

## RESULTS

The Mann-Whitney's two-sample U-test was used to test the significance of differences between treatment and control plots, upper and lower levels, and snowmelt and other periods.

The monthly mean air temperature was highest in August and lowest in February, and the difference between these values was 35°C (Fig. 1). Potential evaporation during the no-snow period (May to October) was highest in June and thereafter gradually decreased.

In the TR plot, the mean $NO_3^-$ concentration in the soil solution was significantly ($p < 0.01$) greater than that of CS and CT plots (Fig. 2). It was applicable to both soil depths. There was no significant difference between the mean $NO_3^-$ concentrations in soil solution in the CS and CT plots. The mean $NO_3^-$ concentration at the upper level (10 cm) was significantly ($p < 0.01$) higher than that of the lower level (40 cm) in the TR plot. There was no significant difference of the mean $NH_4^+$ concentrations in soil solution among plots and between upper and lower depths of soil in each plot.

Monthly mean $NO_3^-$ concentration in soil solution at 10 cm increased during the warm period from July to September in the TR plot (Fig. 3). Monthly mean $NO_3^-$ concentration in soil solution at 40 cm in the TR plot peaked during the period from September to January, and the timing of the peak was different each year. The standard deviation of concentration increased from January 1999. The mean annual concentration of $NO_3^-$ in soil at both depths in the TR plot significantly decreased ($p < 0.05$) from 1998 to 2000. Some data are missing in Fig. 3, because freezing of the sampling tube made soil solution collection impossible.

The soil temperatures at both depths in the TR plot were significantly ($p < 0.01$) higher than those in the two control plots during the growing season (June–October) (Table 1). Soil temperature at the upper level (10 cm) was significantly ($p < 0.05$) higher than that at the lower level (40 cm) in the TR plot. Soil temperatures at both depths in the CS plot were significantly ($p < 0.05$) higher than those in the CT during the growing period.

In the no-snow and snowpack period (from late May to April the following year; NS period), $NO_3^-$ and $Ca^{2+}$ concentrations at both depths in the TR plot were significant higher than those in the control plots ($p < 0.05$ and $p < 0.01$, respectively), but there was no significant difference between $SO_4^{2-}$ concentrations in the three plots (Table 2). In the snowmelt period (from April to late May; SM period), $Ca^{2+}$ concentration in soil at 10-cm depth was higher in the TR plot than in the control plots, but there were no interplot differences in concentrations of other elements. The $SO_4^{2-}$ concentrations in soil at 10 cm in all plots were significantly ($p < 0.01$) higher in the SM period than in the NS period, whereas in the TR plot the $NO_3^-$ concentration at 10 cm was significantly ($p < 0.01$) lower in the SM period than in the NS period. The $NO_3^-$ concentration strongly correlated with $Mg^{2+}$ and $Ca^{2+}$ ($r^2 = 0.889$ and 0.872, respectively) in the TR plot during the NS period, but there was no significant correlation during the SM period.

The mean ion concentration of $NO_3^-$, $SO_4^{2-}$, $Ca^{2+}$, and $Mg^{2+}$ in snowpack water was lower than that in soil solution at all depths in the TR plot. The amount of snowpack water at maximum snow depth was greater in 1999 (Table 3) than in 2000 (data not displayed).

There was a large net output of $NO_3^-$ from soil in the TR plot during the snowmelt period, but there was no net output of $NO_3^-$ from soil in the control plots (Table 4). Both $Mg^{2+}$ and $Ca^{2+}$ were also leached from soil in the control plots during the snowmelt period. The annual net output of $NO_3^-$ at the TR plot was 257 mmol $m^{-2}$ $year^{-1}$, and 145 mmol $m^{-2}$ $period^{-1}$ was leached in the snowmelt period. The contributions of $NO_3^-$, $Mg^{2+}$, and $Ca^{2+}$ net

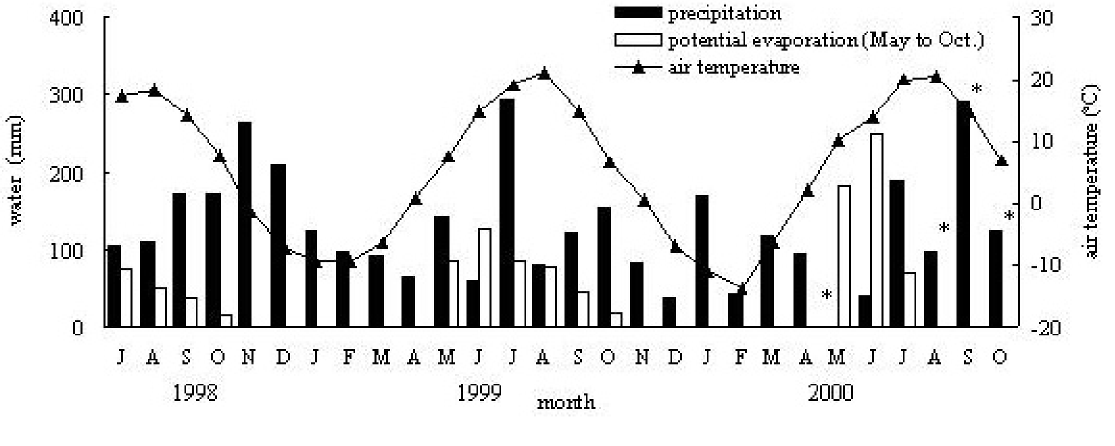

**FIGURE 1**. Monthly total precipitation and mean air temperature during the study period and monthly total evaporation from May to October at an open site adjacent to the study site. Asterisks denote undetermined. From the middle of November to early April the following year, precipitation was snow.

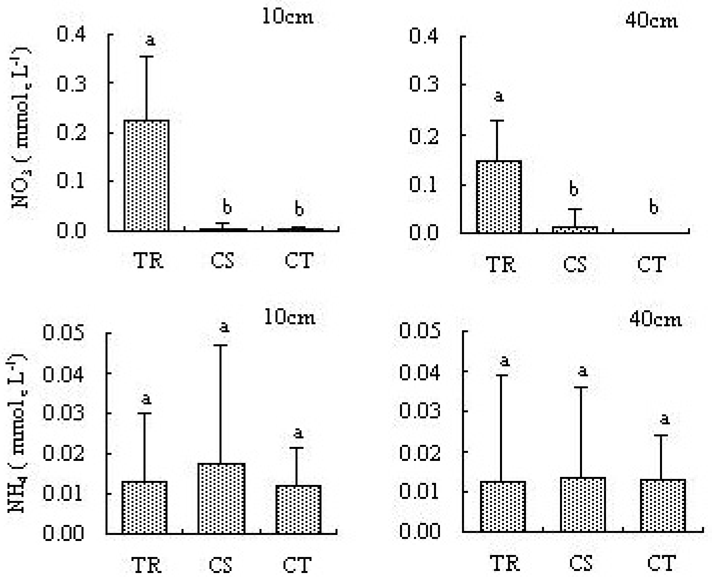

**FIGURE 2.** Mean concentrations of $NO_3^-$ and $NH_4^+$ in soil solution at depths of 10 and 40 cm during 2 years from July 1998 to July 2000. TR: treated; CS: control (sasa); CT: control (tree-sasa). Error bar represents +1 SD for n = 31 – 49. Different letters indicate significant differences ($p < 0.01$).

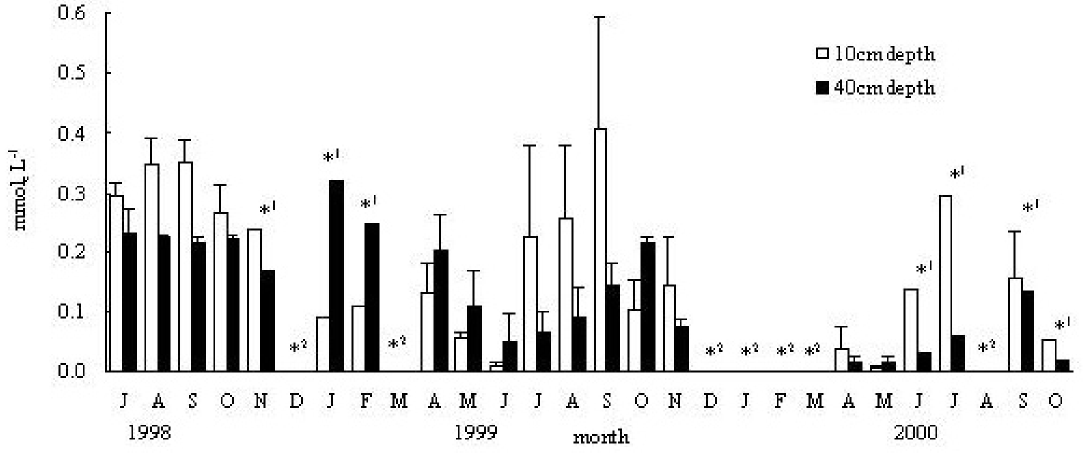

**FIGURE 3.** Mean monthly concentrations of $NO_3^-$ in soil solution at depth of 10 and 40 cm in the TR (treatment) plot. Error bar represents +1 SD for n = 3 – 8. Asterisk 1 (*[1]) indicates inability to calculate the standard deviations due to an insufficient sample number, and asterisk 2 (*[2]) denotes sampling was impossible.

outputs from treated soil during snowmelt to annual fluxes were 57, 89, and 68%, respectively.

## DISCUSSION

### Fluctuation of $NO_3^-$ Concentration Due to Surface Soil Removal

Removal of vegetation and surface soil resulted in a large increase in the mean concentration of $NO_3^-$ in soil solution. Higher $NO_3^-$ concentration in the TR plot was considered the major factor in the decrease of the vegetation uptake due to vegetation removal. In addition, the changes of microbial activity would also be an important factor in this study. The intensive soil preparation, deficient in important substrates for microbial metabolism, would cause the decrease in N immobilization, and it caused the increased $NO_3^-$ into soil[6]. Moreover, the net production of $NO_3^-$ probably increased due to the acceleration of soil N mineralization and nitrification from the increase in soil temperature in the TR plot. The effect of the increase in soil temperature on $NO_3^-$ concentration in exposed mineral soil that is less affected

**TABLE 1**
**Mean Soil Temperatures (°C) at Depths of 10 and 40 cm in Each Plot during the Growing Season (June–October 1999)**

| site | soil depth | |
|---|---|---|
| | 10cm | 40cm |
| treatment | 18.5 [a] | 17.1 [a] |
| control (sasa) | 15.2 [b] | 14.0 [b] |
| control (tree-sasa) | 14.4 [c] | 13.3 [c] |

*Note:* Different letters indicate significant differences between sites.

**TABLE 2**
**Mean Concentrations over a 2-Year Period from July 1998–July 2000 of Major Inorganic Ions and pH in Soil Solution during the Snowmelt Period (from April to late May: SM) and Other Periods (No-Snow and Snowpack, from late May to April the following year: NS)**

| site | soil depth | period | ion concentration ($\mu mol_c$ $L^{-1}$) | | | | | | | | pH | |
|---|---|---|---|---|---|---|---|---|---|---|---|---|
| | | | $NO_3^-$ | | $SO_4^{2-}$ | | $Mg^{2+}$ | | $Ca^{2+}$ | | | |
| TR | 10cm | NS | 254.6 | (120.4) | 27.9 | (14.4) | 56.2 | (20.0) | 117.5 | (53.6) | 5.4 | (0.3) |
| | | SM | 61.8 | (52.6) | 106.9 | (53.1) | 89.2 | (88.0) | 111.2 | (61.1) | 5.7 | (0.2) |
| | 40cm | NS | 153.8 | (81.4) | 21.9 | (7.0) | 39.3 | (17.1) | 74.2 | (36.3) | 5.5 | (0.2) |
| | | SM | 97.2 | (81.1) | 68.7 | (60.1) | 72.1 | (58.1) | 101.3 | (50.0) | 5.6 | (0.1) |
| CS | 10cm | NS | 2.9 | (8.6) | 25.1 | (27.4) | 35.7 | (21.2) | 35.6 | (23.3) | 5.8 | (0.5) |
| | | SM | 13.5 | (11.4) | 98.9 | (58.3) | 77.0 | (80.3) | 54.9 | (26.7) | 5.5 | (0.1) |
| | 40cm | NS | 2.6 | (7.5) | 64.4 | (32.6) | 17.5 | (26.3) | 20.1 | (59.3) | 6.1 | (0.4) |
| | | SM | 50.1 | (79.7) | 60.8 | (34.4) | 61.9 | (39.4) | 42.6 | (19.9) | 5.8 | (0.2) |
| CT | 10cm | NS | 0.9 | (1.6) | 47.6 | (21.4) | 41.0 | (35.9) | 35.8 | (27.5) | 5.3 | (0.4) |
| | | SM | 8.3 | (8.2) | 104.5 | (43.7) | 85.2 | (50.9) | 72.1 | (48.7) | 5.1 | (0.2) |
| | 40cm | NS | 0.6 | (1.2) | 92.8 | (34.2) | 39.7 | (18.7) | 23.0 | (21.7) | 5.7 | (0.4) |
| | | SM | n.d. | | 149.4 | (18.7) | 74.3 | (36.7) | 39.3 | (20.5) | 5.3 | (0.0) |

*Note:* TR: treatment; CS: control (sasa); CT: control (tree-sasa). Values in parentheses are standard deviations.

**TABLE 3**
**Mean Ionic Concentrations and Quantities of Water in the Snowpack in 1999**

| sampling date | water mm | $NO_3^-$ | $SO_4^{2-}$ | $Mg^{2+}$ | $Ca^{2+}$ |
|---|---|---|---|---|---|
| | | $\mu mol_c$ $L^{-1}$ | | | |
| January 7 | 412.3 | 14.2 | 39.3 | 8.7 | 26.8 |
| Feburary 26 | 765.5 | 11.7 | 46.3 | 16.2 | 32.0 |
| May 7 | 297.6 | n.d. | 7.9 | 5.5 | 4.9 |
| average | 491.8 | 13.0 | 31.1 | 10.1 | 21.3 |

*Note:* Not detected concentration denoted as n.d.

**TABLE 4**
**Net Output (Output-Input) of Elements and Water from Rooted Soil (40 cm) during the Snowmelt Period (from April to late May: SM), Other Periods (No-Snow and Snowpack, from late May to April the following year: NS), and Annual (July 1998–July 1999)**

| site | period | date | output water mm | net output water mm | $NO_3^-$ | $NH_4^+$ | $Mg^{2+}$ | $Ca^{2+}$ |
|---|---|---|---|---|---|---|---|---|
| | | | | | mmol $m^{-2}$ $period^{-1}$ | | | |
| TR | NS | 319 | 534 | -246 | 112 | -9 | 20 | 50 |
| | SM | 46 | 900 | 0 | 145 | -10 | 157 | 107 |
| | annual | 365 | 1434 | -246 | 257 | -19 | 177 | 157 |
| CS | NS | 319 | 485 | -296 | -5 | -14 | 0 | -1 |
| | SM | 46 | 1022 | 0 | -4 | 1 | 45 | 26 |
| | annual | 365 | 1506 | -296 | -9 | -13 | 45 | 25 |
| CT | NS | 319 | 356 | -295 | -3 | -6 | 4 | -3 |
| | SM | 46 | 733 | 0 | -10 | -4 | 49 | 6 |
| | annual | 365 | 1089 | -295 | -13 | -9 | 53 | 3 |

*Note:* TR, treatment; CS, control (sasa); CT, control (tree-sasa).

by vegetation uptake before treatment may be considered. The mean $NH_4^+$ concentration was not significantly higher, and the mean $NO_3^-$ concentration was significantly higher at the TR plot than those at the control plots, suggesting the $NH_4^+$ in the TR plot was nitrified immediately.

Although the nitrification process produces two H ions for each $NO_3^-$, pH in soil solution at the upper level in the TR plot was unchanged by year-round treatment. The mean $NO_3^-$ concentration at the upper level was significantly correlated with $Mg^{2+}$ and $Ca^{2+}$ in the no-snow and snowpack period. These results suggested that the H ions were neutralized by cation exchange and soil weathering. In a snowmelt period, there was no significant correlation of $NO_3^-$ with $Mg^{2+}$and $Ca^{2+}$, suggesting that other anions (e.g., $SO_4^{2-}$) contained in the snowpack and soil may also move concurrently with these cations.

The greatest increase in monthly $NO_3^-$ concentration at the upper level occurred in the warm period, while that at the lower level occurred later than that at the upper level (Fig. 3). The decreased evaporation from late summer to autumn may have increased the input water in soil and increased vertical transport of a soil solution of high $NO_3^-$ concentration from the upper level to lower level. Moreover, in the snowpack period, the high concentration of $NO_3^-$ at the lower level in the TR plot resulted from the transport of $NO_3^-$ from the upper to lower levels with a small amount of infiltration of water into soil under the snowpack.

## Effect of Snowmelt on Element Concentration and Budget in Treated Soil

The mean $NO_3^-$ concentrations in the TR plot were significantly high among plots in the no-snow period, which in the snowmelt period decreased to a similar level of those in the control plots. This indicated that the dilution effect by the large amount of snowmelt water whose concentrations were lower than those of soil solutions (Tables 2 and 3). We found that a large amount of $NO_3^-$ was leached from rooted soil at the treated site in the snowmelt period, and it was probably caused by extensive and rapid infiltration of meltwater into the soil. The nitrification at the TR plot in the snowpack and snowmelt period may be small because the microbial activity declined under low temperatures. Therefore, most of the leached $NO_3^-$ in the snowmelt period was produced in the no-snow period, and those were retained in soil under the snowpack and leached by the large amount of meltwater infiltrated into soil in the snowmelt period. Since Rascher et al.[11] suggested that the nitrification under the snowpack may be an important source of $NO_3^-$ in surfacewaters—even during snowmelt at northern forests—more research is needed for nitrification potential under low temperatures.

In the present study, snowmelt lasting for 46 days indicated the rapid leaching of $NO_3^-$ in the TR plot. This rapid rate of $NO_3^-$ leaching occurring in the snowmelt period of low biological activity probably had considerable effects on an annual leaching of $NO_3^-$ in disturbed soil (Table 4). The monthly mean $NO_3^-$ concentration in the soil solution in the TR plot decreased annually. The large amount of leaching of $NO_3^-$ from soil and the dilution of $NO_3^-$ by snowmelt may contribute to the temporal decrease of $NO_3^-$ concentration from 1998 to 2000 (Fig. 3). Moreover, the removal of forest floor and surface soil that are important N sources was the reason for the $NO_3^-$ decrease. Although the reduction in the increased $NO_3^-$ concentration after clear-cut treatment was caused by the increase in the uptake by regenerated vegetation reported in the previous study[2], the vegetation in the treated plot in this study barely recovered in 2 to 4 years after the treatment, and its effect of uptake by vegetation on $NO_3^-$ concentration in soil solution may be small. Effects of the changes in the vegetation absorption and the microbial activity on the fluctuation of $NO_3^-$ will increase along with the regeneration of the vegetation. Therefore, continued observation is needed including the temporal fluctuation of

$NO_3^-$ in soil, the vegetation uptake of $NO_3^-$, and the microbial activity.

## CONCLUSION

In a snow-dominated forest, removal of the vegetation and the surface soil resulted in an increase in $NO_3^-$ concentration in the soil solution and a large amount of $NO_3^-$ leaching from soil. The increase in $NO_3^-$ concentration in the soil solution was likely to have been because of decreased vegetation uptake, greater net mineralization, and nitrification rate resulting from surface soil removal and soil temperature increase. Acidification of soil following nitrification did not occur in the treated plot because of proton neutralizing by cation exchange. $NO_3^-$ produced at the surface soil was transported to the deeper soil by the water movement in the soil, particularly from autumn to early winter. In the snowmelt period, when the soil temperature and rate of nitrification were lower compared with that in the no-snow period, a large amount of meltwater infiltrated into the soil and caused a rapid, sizeable discharge of $NO_3^-$.

## ACKNOWLEDGEMENT

We are very grateful to the staff in the Uryu Experimental Forest for the field assistance and providing the data of forest stand measurement and meteorology. We also acknowledge the reviewers for substantially reviewing this manuscript.

## References

1. Aber, J.D., Nadelhoffer, K.J., Sterdler, P., and Mellilo, J.M. (1989) N saturation in northern forest ecosystems. *BioScience* **39**, 378–386.
2. Bormann, F.H. and Likens, G.E. (1979) *Pattern and Process in a Forested Ecosystem*. Springer-Verlag, New York. 253 p.
3. Hornbeck, J.W., Martin, C.W., Pierce, R.S., Bormann, F.H., Likens, G.E., and Eaton, J.S. (1986) Clearcutting northern hardwoods: effects on hydrologic and nutrient ion budgets. *For. Sci.* **32**, 667–686.
4. Edmonds, R.L. and McColl, J.G. (1989) Effects of forest management on soil N in Pinus radiata stands in the Australian Capital Territory. *For. Ecol. Manage*. **29**, 199–212.
5. Frazer, D.W., McColl, J.G., and Powers, R.F. (1990) Soil N mineralization in a clearcutting chronosequence in a northern California conifer forest. *Soil Sci. Soc. Am. J.* **54**, 1145–1152.
6. Vitousek, P.M. and Matson, P.A. (1985) Disturbance, N availability, and N losses in an intensively managed loblolly pine plantation. *Ecology* **66**, 1360–1376.
7. Williams, M.W. and Melack, J.M. (1991) Solute chemistry of snowmelt and runoff in an alpine basin, Sierra Nevada. *Water Resour. Res*. **27**, 1575–1588.
8. Stottlemyer, R. and Toczydlowski, D. (1996) Modification of snowmelt chemistry by forest floor and mineral soil, Northern Michigan. *J. Environ. Qual.* **25**, 828–836.
9. Ozawa, M., Shibata, H., Satoh, F., and Sasa, K. (2001) Annual element budget of soil in snow-dominated forested ecosystem. *Water Air Soil Pollut.* in press.
10. Nomura, M., Satoh, F., Ashiya, D., and Masumoto, H (1999) Research Bulletin of the Hokkaido University Forests 56, 11, Hokkaido University, Sapporo (in Japanese with English summary).
11. Rascher, C.M., Driscoll, C.T., and Peters, N.E. (1987) Concentration and flux of solutes from snow and forest floor during snowmelt in the west-central Adirondack region of New York. *Biogeochemistry* **3**, 209–224.

| | | |
|---|---|---|
| Received: | July | 16, 2001 |
| Revised: | October | 8, 2001 |
| Accepted: | October | 15, 2001 |
| Published: | November | 22, 2001 |
